# Over-expressed microRNA-181a reduces glomerular sclerosis and renal tubular epithelial injury in rats with chronic kidney disease via down-regulation of the TLR/NF-κB pathway by binding to CRY1

**DOI:** 10.1186/s10020-018-0045-2

**Published:** 2018-09-18

**Authors:** Lei Liu, Xin-Lu Pang, Wen-Jun Shang, Hong-Chang Xie, Jun-Xiang Wang, Gui-Wen Feng

**Affiliations:** Department of Kidney Transplantation, The First Affiliated Hospital of Zhengzhou University, No. 1, Jianshe Road, Erqi District, Zhengzhou, 450052 Henan Province People’s Republic of China

**Keywords:** MicroRNA-181a, CRY1 gene, The TLR/NF-κB pathway, Chronic kidney disease, Glomerular sclerosis, Renal tubular epithelial injury

## Abstract

**Background:**

MicroRNAs (miRNAs) contribute to the progression of chronic kidney disease (CKD) by regulating renal homeostasis. This study explored the effects of miR-181a on CKD through the Toll-like receptor (TLR)/nuclear factor-kappa B (NF-κB) pathway by binding to CRY1.

**Methods:**

Seventy male rats were selected and assigned into specific groups: miR-181a mimic, miR-181a inhibitor, and siRNA against CRY1, with each group undergoing different treatments to investigate many different outcomes. First, 24-h urinary protein was measured. ELISA was used to determine the serum levels of SOD, ROS, MDA, IL-1β, IL-6, and TNF-α. Biochemical tests for renal function were performed to measure albumin, uric acid, and urea in urine and urea nitrogen and creatinine in serum. The glomerulosclerosis index (GSI) and renal tubular epithelial (RTE) cell apoptosis were detected using PASM staining and TUNEL staining, respectively. Finally, RT-qPCR and western blot were done to determine miR-181a, CRY1, TLR2, TLR4, and NF-κB expression.

**Results:**

CRY1 is the target gene of miR-181a, according to a target prediction program and luciferase assay. Rats diagnosed with CKD presented increases in 24-h urinary protein; GSI; RTE cell apoptosis rate; serum ROS, MDA, IL-1β, IL-6, and TNF-α; and CRY1, TLR2, TLR4, and NF-κB expression, as well as decreases in SOD level and miR-181a expression. Following transfection with either the miR-181a mimic or si-CRY1, 24-h urinary protein, renal damage, GSI, and cell apoptosis rate were all decreased. In addition, the overexpression of miR-181a or inhibition of CRY1 alleviated the degree of kidney injury through suppression of the TLR/NF-κB pathway.

**Conclusion:**

miR-181a alleviates both GS and RTE injury in CKD via the down-regulation of the CRY1 gene and the TLR/NF-κB pathway.

## Background

Chronic kidney disease (CKD) is a highly prevalent public health problem, and its incidence is rapidly increasing worldwide. Approximately 500 million people worldwide suffer from CKD, and the progression of the disease is much faster in the male population when compared with females (Gandolfo et al. 2004; Sampaio-Maia et al. 2016). CKD is characterized by certain adverse outcomes, including cardiovascular disease (CVD), kidney function failure, and premature death (Levey et al. 2005). The major histological findings in CKD patients are often glomerulosclerosis and tubulointerstitial fibrosis (Munoz-Felix et al. 2014). CKD can be classified based on the stages of disease severity, which are evaluated by measuring the body’s glomerular filtration rate and albuminuria, as well as making a clinical diagnosis (Levey and Coresh 2012). Although the occurrence of CKD is age-independent, individuals over the age of 75 years are more susceptible to CKD (O’Hare et al. 2007). Significant advances have been made in controlling the progression of CVD, but the incidence and prevalence of CKD are still alarmingly high rates (Gaddam et al. 2016). Therefore, better studies and observations are necessary to provide a better understanding, diagnosis, and prognosis of CKD. A recent breakthrough has been the discovery that microRNAs (miRNAs), small non-coding RNA molecules, are involved in cancer (Lorenzen et al. 2011).

A member of the miR-181 family, miR-181a, functions as a mitochondrial miRNA in human kidneys (Marques et al. 2015; Su et al. 2015). Overexpression of miR-181a could very well enhance cell proliferation as well as reduce apoptosis of clear cell renal cell carcinoma via the down-regulation of KLF6 expression (Lei et al. 2017). Furthermore, Na et al. revealed that miR-181d could potentially target circadian rhythm genes, including cryptochrome1 (CRY1) and others (Na et al. 2009). CRY1, a circadian clock gene, is able to maintain its rhythmicity by inhibiting BMAL1/Clock transcriptional activity, thereby exerting pro-inflammation functions (Lee et al. 2013; Yang et al. 2015). For instance, down-regulating CRY activates pro-inflammatory cytokines via the nuclear factor-kappa B (NF-κB) pathway (Narasimamurthy et al. 2012). Up-regulated CRY1 could also potentially reduce the development of atherosclerosis by regulating the Toll-like receptor (TLR)/NF-κB pathway (Yang et al. 2015). The activation of the TLR2-MyD88-NF-κB pathway has been associated with the tubulointerstitial inflammation in CKD, further suggesting that the TLR/NF-κB pathway could be used as a potential target treatment for CKD (Ding et al. 2015).

Based on the aforementioned findings, we hypothesized that miR-181a, CRY1, and the TLR/NF-κB pathway are involved in the progression, incidence, and treatment of CKD. Few researchers have investigated the underlying mechanism of miR-181a in CKD via the TLR/NF-κB pathway. Therefore, we established a rat model to explore whether miR-181a influenced CKD by way of the TLR/NF-κB pathway via regulation of the CRY1 gene.

## Materials

### Ethics statement

All animal experiments in this study were conducted in accordance with guidelines for the use and care of laboratory animals.

### Model establishment and transfection

A total of 70 male Sprague-Dawley (SD) rats aged 7 weeks and weighing approximately 210 ± 20 g were purchased from the Experimental Animal Centre of Southern Medical University. The rats were then randomly divided into two groups, with ten rats for the normal group and the remaining 60 rats for the CKD group, which were then used for the establishment of the CKD rat models through an intravenous injection of adriamycin into the tail vein (3 mg/kg) (Ding et al. 2014). Following 1 week of administration of adriamycin, the rats in the CKD group were classified into the following subcategories: blank (transfected without any sequences), negative control (NC) (transfected with fluorescence-labeled NC plasmids), miR-181a mimic (transfected with fluorescence-labeled miR-181a mimic plasmids), miR-181a inhibitor (transfected with fluorescence-labeled miR-181a inhibitor plasmids), si-CRY1 (transfected with fluorescence-labeled si-CRY1 plasmids), and miR-181a inhibitor + si-CRY1 (transfected with fluorescence-labeled miR-181a inhibitor + si-CRY1 plasmids). All transfected plasmids were designed and purchased by the Sangon Biotech Co., Ltd. (Shanghai, China). The rats in all CKD groups were injected with 0.002 μg plasmids via the tail vein, while the rats in the normal group were injected with an equal volume of normal saline via their tail vein. We collected 24-h urine samples from rats in each group at the end of the 4th week, along with their peripheral blood, which was extracted from the tail of rats to prepare the serum. The serum prepared from the peripheral blood was finally stored at − 20 °C for later use. The rats were then sacrificed following successful collection of the serum, and the kidneys were collected soon after and preserved. The renal capsule was then peeled off the kidney tissues, with some of the kidney tissues being preserved in 4% paraformaldehyde for cryostat sectioning, fluorescence microscopy detection, and terminal deoxyribonucleotidyl transferase (TDT)-mediated dUTP-digoxigenin nick end labelling (TUNEL) staining. Some of the cortex renis that remained was then placed in a 10% formaldehyde for pathological examination. The remaining kidney tissues were all preserved in liquid nitrogen to be used in later experiments.

### Transfection of plasmids

Kidney tissues preserved with 4% paraformaldehyde overnight were then removed from their preservative and dehydrated using a sucrose solution until the kidney tissue settlement was noticeable at the bottom of the container. The tissues were then sliced into 45 μm sections at − 22 °C using a pre-cooled freezing microtome (HM525, SeaWorld Equipment Co., Ltd., Beijing, China), followed by dicing an appropriate amount of kidney tissue and soaking the pieces in a 6-well plate in ice-cold phosphate-buffered saline (PBS). The pathological slides were then treated with pre-gelatin to prevent tissue from falling off, sealed with anti-fluorescence quenching agents, and observed under a fluorescence microscope. Each field of vision was then photographed in both the bright field (natural light) and dark field (without natural light). The experiment was repeated 3 times.

### Detection of 24-h urinary protein

Following the 4th week of observation, the 24-h urine was collected and its volume was recorded after all of the rats had been placed in a metal metabolic cage. Next, 5 mL of the collected 24-h urine was centrifuged at 3000 r/min to remove the sediment, stored in a freezer at 4 °C, and finally sent away to the laboratory for the quantitative determination of urinary protein. This experiment was repeated three times.

### Enzyme-linked immunosorbent assay (ELISA)

Following the urinalysis, serum samples were centrifuged at 3000 r/min and the supernatant was taken. According to the instructions provided by the ELISA kit (YQ, Imun Bio-Tech Co., Ltd., Beijing, China), the contents of superoxide dismutase (SOD, E-33106), malondialdehyde (MDA, 10417R), reactive oxygen species (ROS, 10256R) (all from Shanghai Yuanye Bio-Technology Co., Ltd., Shanghai, China), interleukin-1β (IL-1β, E-30418), interleukin-6 (IL-6, E-30644), and tumor necrosis factor (TNF-α, E-30633) (all from Xiamen Huijia Biotechnology Co., Ltd., Xiamen, China) were all determined. All kits were purchased from Abcam (Cambridge, MA, USA). The optical density (OD) value of each well was evaluated at 450 nm in a microplate reader (BS-1101, Detie Laboratory Equipment Co., Ltd., Nanjing, China) after blank-control wells were set at zero. The experiments for each group were repeated three times.

### Detection of renal functions

Urine samples were collected from each group to measure urea, uric acid, and albumin. After the urine had been collected, the rats were sacrificed via neck dislocation, allowing for the blood to be collected, and centrifuged at 2000 r/min at 4 °C for 10 min. The serum was then extracted, and serum creatinine and urea nitrogen were measured.

### Hematoxylin-eosin (HE) staining

The kidney tissues were placed in a 10% neutral formaldehyde solution for over 24 h, after which they were rinsed under running tap water, conventionally dehydrated for 1 min, cleaned in xylene twice for 5 min, soaked in and embedded with paraffin, placed in the paraffin model, and finally left to cool on a cold bench. The paraffin sections were then stained using a hematoxylin (PT001, Shanghai Bogoo Science & Technology Co., Ltd., Shanghai, China) staining method at room temperature for a total of 10 min, followed by washing under running water for 30–60 s. The sections were then placed in eosin (0001-H, Beijing XinHuaLvYuan Science and Technology Co., Ltd., Beijing, China) at room temperature for 1 min, followed by dehydration in a gradient of ethanol and clearing in xylene I and xylene II (GD-RY1215–12, Shanghai Guduo Science and Technology Co., Ltd., Shanghai, China) twice for 1 min. After being sealed with neutral balsam, we observed the pathological changes of kidney tissues under a Caikon fluorescence microscope (PrimoStar iLED, Bioresearch & Technology Co., Ltd., Beijing, China). Glomerular hypertrophy was confirmed by measuring the mean glomerular perimeter of the 20 largest glomeruli (Uil et al. 2018). The experiment was repeated three times.

### Periodic acid of Schiff-methenamine (PASM) staining

Paraffin sections of each group were dewaxed in water, oxidized with a 0.5% periodic acid for 30 min, and washed under both tap water and distilled water. After oxidizing with a 10% chromic acid for 20 min, the sections were then rinsed with distilled water, fixed in a 1% sodium sulfite solution for 30 s, washed with distilled water, and immersed under a hexamine-silver solution (JR00477, Shanghai Junrui Biotechnology Co., Ltd., Shanghai, China) for 30 min. The sections were then left to stand at a temperature of 65 °C for a total of 4 min for a water bath, after which they were removed and washed with distilled water and measured under a microscope until the black precipitate appeared in the glomerular capillary basement membrane. Following the appearance of the black precipitate, the sections were colored using a 0.1% auric chloride solution (G810493-5 g, Shanghai Chaoyan Biotechnology Co., Ltd., Shanghai, China) for 30 s, washed under running water for 10 min, and stained with hematoxylin and 0.5% eosin for a total of 3 min. After completing a conventional dehydration cycle, the sections were cleared, sealed, and observed (Other reagents were all prepared by our laboratory). Pathological changes of glomeruli were observed under a microscope, including the collapse or dissolution of the glomerular capillary network, formation of micro-thrombi in the renal capillary lumen, fracture in the renal capsule, and fibrin exuded and sclerotized in glomeruli. The sections were then further observed under the microscope (400 ×) in 20 random fields per slice. Glomerular basement membrane, reticular fibers, and type IV collagen were all discovered to be black, the nucleus was both blue and black, and the background was pink. The degree of the glomerular lesion was evaluated according to the glomerulosclerosis index (GSI). According to the proportion of GS accounting for the glomeruli, GSI was divided into 0–4 levels: grade 0, normal; grade 1, GSI of < 25%; grade 2, GSI of 25–50%; grade 3, GSI of 50–75%; grade 4, GSI of 75–100%. With the grade scoring determined, the GSI was then calculated using the following formula: GSI = [(1 × N1) + (2 × N2) + (3 × N3) + (4 × N4) / NT] × 100%, where N1-N4 indicate the numbers of glomeruli in grades 1–4 of glomerulosclerosis, respectively. Each experiment was conducted a total of 3 times.

### Masson staining

Following the determination of the glomerulosclerosis index, the sections were dewaxed, dehydrated, stained with hematoxylin (PT001, Shanghai Bogoo Science & Technology Co., Ltd., Shanghai, China) for 8 min, and rinsed using tap water. After being placed in a mixture of 1% Ponceau (HL12202, Shanghai Haling Biotechnology Co., Ltd., Shanghai, China) and 1% fuchsin solution (HPBIO-SJ820, Shanghai Hepeng Biotechnology Co., Ltd., Shanghai, China) for 40 min, the reaction was terminated by addition of 1% glacial acetic acid and 1% molybdic acid solution, followed by addition of a mixture of 1% brilliant green and 1% phosphomolybdic acid in tap water. The sections were then conventionally dehydrated and mounted along with a neutral balata. The degree of the tubulointerstitial lesions was determined by observing both the normal and atrophic states of the renal tubules, as well as the degree of the inflammatory cell infiltration (other reagents were prepared by our laboratory). As a result, 20 fields of vision in each section were randomly selected to be observed under the microscope (400 ×). The basement membrane and the collagen fibers were stained either blue or green, the immune complex was stained red, and the nuclei were stained blue-brown. According to the ratios between the positive area of inflammatory cell infiltration, interstitial fibrosis, and the areas of glomeruli and renal tubules, the degree of the tubulointerstitial lesions was graded on a scale ranging between 0 and 4 points (0 points = negative area; 1 point = positive area < 10%; 2 points = 10–25% involvement of positive area; 3 points = 25–50% involvement of positive area; 4 points = positive area > 50%). Each experiment was conducted 3 times.

### TUNEL staining

For the TUNEL staining process, kidney tissues were embedded in paraffin, sectioned, and stained according to the instructions provided by the TUNEL kit (40302ES20, Yeasen, USA). The nuclei of the apoptotic cells that were stained presented a brownish-yellow color, while the nuclei of normal cells were stained blue. The number of apoptotic cells in each renal field of vision was calculated using a digital pathologic slice scanner (6,504,523,001, Roche Diagnostics Ltd., Shanghai, China) from fields observed under a microscope (200 ×). Following the random selection of five fields of vision of each kidney section, the percentage of positive cells in each field was calculated, as was the average of all fields. The average proportion was the apoptotic index (AI). Each experiment was conducted a total of three times.

### Dual luciferase reporter gene assay

The targeting regulatory relationship between miR-181a and the CRY1 gene was predicted with the bioinformatics prediction website http://www.microRNA.org, and a dual luciferase reporter gene assay was carried out to verify whether CRY1 was indeed a target gene of miR-181a. According to the binding sequence of the 3’UTR region of the combination of CRY1 mRNA and miR-181a, the target sequences as well as the mutation sequences were designed, and the target sequences were chemically synthesized; simultaneously, Xho I and Not I were added at the respective ends of the sequences. The fragments were cloned into the PUC57 vector. After identifying the positive clones, the recombinant plasmids were identified via DNA sequencing, after which they were further sub-cloned into the psiCHECK-2 vector and transformed into *Escherichia coli* DH5α cells to amplify the plasmids. The plasmids were then extracted following amplification according to the instructions provided by the Omega kit (D1100-50 T, Beijing Solarbio Science & Technology Co., Ltd., Beijing, China). Next, the cells were inoculated into a 6-well plate at a density rate of 2 × 10^5^ cells per well. Following the adherence of the cells, they were transfected using the aforementioned inoculation method, cultured for a total of 48 h, and collected. The effect of miR-181a on the luciferase activity of the CRY1 3’-UTR in cells was examined according to the method provided by the dual luciferase gene assay kit (D0010, Beijing Solarbio Science & Technology Co., Ltd., Beijing, China). The fluorescence intensity was measured using a Glomax 20/20 luminometer fluorescence detector (E5311, Shaanxi Zhongmei Biotechnology Co., Ltd., Shaanxi, China). The aforementioned procedures were conducted in triplicate.

### Reverse transcription quantitative polymerase chain reaction (RT-qPCR)

The total RNA was extracted from kidney tissues by using the miRNeasy Mini Kit (217,004, QIAGEN, Germany), and the primers for miR-181a, CRY1, TLR2, TLR4, NF-κB, U6, and β-actin were all designed and simultaneously synthesized by TaKaRa Biotechnology Co. Ltd. (Liaoning, China) (Table 1). The sample RNA was reverse-transcribed into cDNA in a reaction system of 10 μL according to the instructions provided by PrimeScript TM RT reagent kit (RR036A, TaKaRa Biotechnology Co. Ltd., Liaoning, China). The conditions for the reaction system were reverse transcription at 37 °C 3 times (15 min each) and inactivation of reverse transcriptase at 85 °C for 5 s. Subsequently, we performed RT-qPCR according to the instructions provided by the SYBR® Premix Ex TaqTM II Kit (RR820A, TaKaRa Biotechnology Co. Ltd., Liaoning, China) using an ABI 7500 PCR machine (7500, ABI, USA). The PCR mix was 25 μL of SYBR® Premix Ex Taq™ II (2 ×), 2 μL of forward primer, 2 μL of reverse primer, 1 μL of ROX Reference Dye (50 ×), 4 μL of DNA template, and 16 μL of ddH_2_O. The reaction conditions consisted of pre-denaturation at 95 °C for 30 s, 40 cycles of denaturation at 95 °C for 5 s, annealing at 60 °C for 30 s, and extension at 60 °C for 30 s. Afterwards, with 2 μg of total RNA as the template, U6 as the internal reference for miR-181a expression, and β-actin as the internal reference for the expression of CRY1, TLR2, TLR4, and NF-κB, the relative expression of miR-181a, CRY1, TLR2, TLR4, and NF-κB was analyzed by the 2^-△△Ct^ method, where △△Ct = Ct _target gene_ - Ct _internal reference_. Each experiment was conducted in triplicate.

**Table 1:** Primer sequences for RT-qPCR

Genes	Primer sequences
miR-181a	F: 5’-GGGCAGCCTTAAGAGGA-3′
	R: 5’-CAGTGCGTGTCGTGGA-3′
CRY1	F: 5’-TCAGTTGGGAAGAAGGGATG-3′
	R: 5’-CATTGGGGTCTGTCCTCCTA-3′
TLR2	F: 5’-CGCTTCCTGAACTTGTCC-3′
	R: 5’-GGTTGTCACCTGCTTCCA-3′
TLR4	F: 5’-CTATCATCAGTGTATCGGTG-3′
	R: 5’-CAGTCCTCATTCTGGCTC-3′
NF-κB	F: 5’-ATTCAACAGATATACCACTGTCAAC-3′
	R: 5’-TGTTCTTCTCACTGGAGGCA-3′
U6	F: 5’-GCTTCGGCAGCATATACTAAAAT-3′
	R: 5’-CGCTTCACGAATTGTCATTGCGT-3′
β-actin	F: 5’-AGCCATGTACGTAGCCATCC-3′
	R: 5’-CTCTCAGCTGTGGTGGTGAA-3’

Notes: *RT-qPCR* reverse transcription quantitative polymerase chain reaction, *miR-181a* microRNA-181a, *CRY1* cryptochrome 1, *TLR2* toll-like receptor 2, *TLR4* toll-like receptor 4, *NF-κB* nuclear factor-kappa B, *F* forward, *R* reverse

### Western blot assay

The RIPA Kit (R0010, Beijing Solarbio Science & Technology Co., Ltd., Beijing, China) was used to extract total protein from fresh tissues, and the bicinchoninic acid (BCA) kit (G3522–1, Guangzhou JBCBIO Technologies Inc., Guangzhou, China) was used to determine the protein concentration. After protein separation with polyacrylamide gel electrophoresis (PAGE), the protein was transferred onto nitrocellulose (NC) membranes. The membranes were blocked using 5% bovine serum albumin (BSA) at room temperature for 1 h and incubated at 4 °C overnight with rabbit anti-rat primary antibodies (CRY1, ab3518, 1:2000; TLR2, ab213676, 1:800; TLR4, ab83444, 1 μg/ml; NF-κB, ab31432, 1:800). All of the antibodies were purchased from Abcam Inc., Cambridge, MA, USA. After washing with a PBS solution 5 times (5 min per wash), the membrane was incubated with a horseradish peroxidase (HRP)-labeled goat anti-IgG (1:5000, Beijing Zhongshan Biotechnology Co., Ltd., Beijing, China) and developed using an electrochemiluminescence (ECL) solution (WBKLS0500, Pierce, Rockford, IL, USA) for 1 min. Following the removal of the liquid, the membrane was covered with plastic wrap and exposed to an X-ray film (36209ES01, Qiancheng Biology Co., Ltd., Shanghai, China) for further observation. A quantitative protein analysis was conducted based on the ratio between the gray value of each protein and the gray value of β-actin using ImageJ software 1.48 μ (National Institutes of Health). Lastly, the membrane was developed in a darkroom and photographed. Each experiment was conducted in triplicate.

### Statistical analysis

The statistical analysis was performed using the Statistical Package for the Social Sciences (SPSS) 21.0 software (IBM Corp. Armonk, NY, USA). The measurement data are expressed as the mean ± standard deviation. Differences between the two groups were compared using the *t*-test, while differences among multiple groups were compared using one-way analysis of variance (ANOVA). *p* < 0.05 was considered to be statistically significant.

## Results

### The plasmid in each group was successfully transfected

**Fig. 1:**
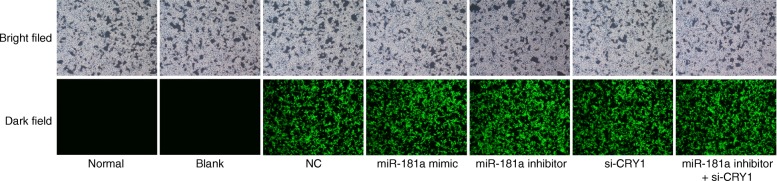
Fluorescence microscopy image of kidney tissue in rats (× 400) reveals that the plasmid in each group was successfully transfected. Notes: The images were photographed in the bright field (natural light) and dark field (without natural light). miR-181a, microRNA-181a; CRY1, cryptochrome 1; CKD, chronic kidney disease; NC, negative control

Green fluorescence was observed in each group under a standard microscope. The results (Fig. 1) revealed no obvious fluorescence in the normal and blank groups, while there was obvious green fluorescence in the NC, miR-181a mimic, miR-181a inhibitor, si-CRY1, and miR-181a mimic + si-CRY1 groups, indicating that the plasmid was successfully transfected in all seven groups.

### Up-regulated miR-181a or down-regulated CRY1 reduces urinary protein levels of CKD

**Fig. 2:**
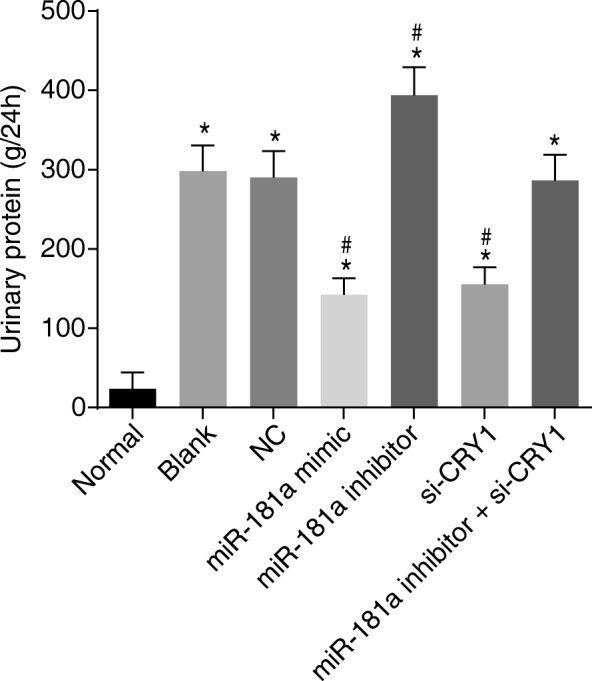
Bar graph shows the 24-h urinary protein level at the end of the 4th week after transfection in each group, demonstrating that up-regulated miR-181a or down-regulated CRY1 leads to the reduction of urinary protein levels in CKD. Notes: ^*^, *p* < 0.05 vs. the normal group; ^#^, *p* < 0.05 vs. the blank and NC groups; the data are presented as the mean ± standard deviation, analyzed by one-way ANOVA; the experiment was independently repeated three times; CRY1, cryptochrome 1; miR-181a, microRNA-181a; CKD, chronic kidney disease; NC, negative control

As seen in Fig. 2, the 24-h urinary protein at the end of the 4th week following transfection had significantly increased among the 6 CKD groups compared with the normal group (*p* < 0.05). No significant differences were observed in the 24-h urinary protein at the end of the 4th week following transfection between the blank and NC groups (*p* > 0.05). In comparison with the blank and NC groups, the 24-h urinary protein at the end of the 4th week following transfection had significantly declined in the miR-181a mimic and si-CRY1 groups, while it was elevated in the miR-181a inhibitor group (*p* < 0.05). However, 24-h urinary protein was not significantly different in the miR-181a inhibitor + si-CRY1 group (*p* > 0.05). These results indicate that up-regulation of miR-181a or down-regulation of CRY1 expression reduces urinary protein in CKD.

### Up-regulated miR-181a or down-regulated CRY1 ameliorates renal damage in CKD

The biochemical tests of renal function (Tables 2 and 3) revealed significant increases in urine albumin, urine uric acid, urine urea, serum urea nitrogen, and serum creatinine at the end of the 4th week in the 6 CKD groups in comparison with the normal group (all *p* < 0.05). Again, no significant differences in the aforementioned indicators between the blank and NC groups were detected (all *p* > 0.05). When comparing the blank and NC groups, the contents of urine albumin, urine uric acid, urine urea, serum urea nitrogen, and serum creatinine in the miR-181a mimic group and si-CRY1 group all showed a significant decrease at the end of the 4th week following transfection, whereas they were all significantly increased in the miR-181a inhibitor group at the end of the 4th week after transfection (*p* < 0.05). There was no significant difference in the content of albumin, uric acid, urea, serum urea nitrogen, or serum creatinine in the miR-181a inhibitor + si-CRY1 group at the end of the 4th week following transfection (all *p* > 0.05) (Tables 2 and 3). The aforementioned findings suggest that the up-regulation of miR-181a or down-regulation of CRY1 reduces the degree of renal damage in CDK.

**Table 2:** Upregulation of miR-181a or downregulation of CRY1 decreases the contents of urine albumin, uric acid, urea of CKD rats (*n* = 10)

Group	Urea (mmol/L)	Uric acid (μmol/L)	Albumin (g/L)
Normal	4.53 ± 0.75	152.32 ± 23.11	34.24 ± 5.12
Blank	48.16 ± 7.54^*^	256.71 ± 33.28^*^	20.97 ± 3.24^*^
NC	47.28 ± 7.23^*^	251.37 ± 32.14^*^	20.07 ± 3.13^*^
MiR-181a mimic	14.76 ± 2.31^*#^	193.48 ± 27.12^*#^	26.14 ± 4.12^*#^
MiR-181a inhibitor	67.82 ± 10.09^*#^	303.45 ± 33.23^*#^	13.92 ± 2.34^*#^
si-CRY1	14.24 ± 2.13^*#^	201.64 ± 25.96^*#^	28.18 ± 4.31^*#^
MiR-181a inhibitor + si-CRY1	46.82 ± 7.22^*^	248.39 ± 31.42^*^	23.26 ± 3.56^*^

Note: ^*^, *p* < 0.05 vs. the normal group; ^#^, *p* < 0.05 vs. the blank and NC groups; the data are presented as mean ± standard deviation and analyzed by one-way ANOVA.; the experiment was independently repeated three times

*NC* negative control, *miR-181a* microRNA-181a, *CRY1* cryptochrome 1, *CKD* chronic kidney disease

**Table 3:** Upregulation of miR-181a or downregulation of CRY1 decreases the contents of serum urea nitrogen and serum creatinine of CKD rats (*n* = 10)

Group	Serum urea nitrogen (mmol/L)	Serum creatinine (mmol/L)
Normal	3.47 ± 0.45	54.28 ± 8.34
Blank	8.39 ± 1.34^*^	94.39 ± 11.32^*^
NC	8.25 ± 1.24^*^	96.24 ± 11.34^*^
MiR-181a mimic	6.14 ± 0.83^*#^	70.73 ± 6.78^*#^
MiR-181a inhibitor	12.31 ± 2.12^*#^	113.65 ± 12.89^*#^
si-CRY1	6.06 ± 0.83^*#^	71.07 ± 6.75^*#^
MiR-181a inhibitor + si-CRY1	8.17 ± 1.23^*^	90.21 ± 11.31^*^

Note: ^*^, *p* < 0.05 vs. the normal group; ^#^, *p* < 0.05 vs. the blank and NC groups; the data are presented as mean ± standard deviation and analyzed by one-way ANOVA.; the experiment was independently repeated three times

*NC* negative control, *miR-181a* microRNA-181a, *CRY1* cryptochrome 1, *CKD* chronic kidney disease

### Up-regulated miR-181a or down-regulated CRY1 inhibits inflammatory infiltration and oxidation

**Fig. 3:**
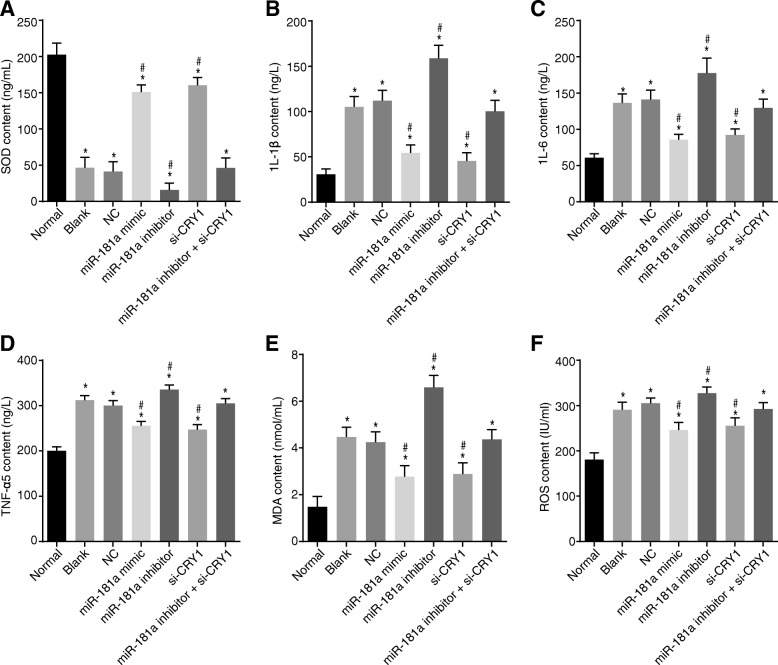
ELISA indicates that serum SOD, ROS, MDA, IL-1β, and IL-6 are decreased while TNF-α is increased after transfection of miR-181a or si-CRY1. Notes: Panel **a**, histogram of SOD; Panel **b**, histogram of IL-1β; Panel **c**, histogram of IL-6; Panel **d**, histogram of TNF-α; Panel **e**, histogram of MDA; Panel **f**, histogram of ROS; ^*^, *p* < 0.05 vs. the normal group; ^#^, *p* < 0.05 vs. the blank and NC groups; the data are presented as the mean ± standard deviation, analyzed by one-way ANOVA; the experiment was independently repeated three times; NC, negative control; miR-181a, microRNA-181a; CRY1, cryptochrome 1; SOD, superoxide dismutase; MDA, malondialdehyde; ROS, reactive oxygen; IL-6, interleukin-6; IL-1β, interleukin-1β; TNF-α, tumor necrosis factor-α

The effects of miR-181a on inflammatory and oxidative conditions were assessed using an ELISA test. As illustrated in Fig. 3, the results showed that, compared with the normal group, ROS, MDA, IL-1β, IL-6, and TNF-α were up-regulated and SOD was down-regulated in the 6 CKD groups (*p* < 0.05). There were no significant differences in the previous parameters between the blank and NC groups (*p* > 0.05). In comparison with both the blank and NC groups, the miR-181a mimic and si-CRY1 groups had decreased ROS, MDA, IL-1β, IL-6, and TNF-α and increased SOD, while the miR-181a inhibitor group showed great elevation in the former values but a decline in SOD (all *p* < 0.05). There was no significant difference in ROS, MDA, IL-1β, IL-6, TNF-α, or SOD in the miR-181a inhibitor + si-CRY1 group compared with the blank and NC groups (*p* > 0.05). These results suggest that up-regulating miR-181a or down-regulating CRY1 expression suppresses both the inflammatory infiltration and oxidation.

### Up-regulated miR-181a or down-regulated CRY1 reduces the degree of CKD

**Fig. 4:**
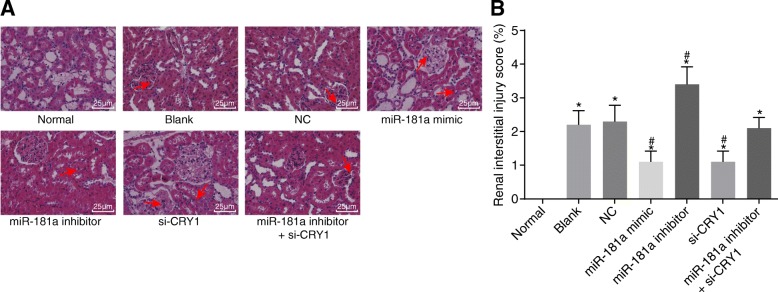
HE staining of kidney tissues (× 400) reveals that the up-regulation of miR-181a or down-regulation of CRY1 reduces the degree of CKD. Notes: the arrows indicate glomeruli; **a** HE staining results in renal tissues (x 400); **b** renal interstitial lesion score. ^*^, *p* < 0.05 vs. the normal group; ^#^, *p* < 0.05 vs. the blank and NC groups; the data are presented as the mean ± standard deviation, analyzed by one-way ANOVA; the experiment was independently repeated three times; HE, hematoxylin-eosin; NC, negative control; miR-181a, microRNA-181a; CRY1, cryptochrome 1

The transfection of the plasmids was observed using the HE staining method. As seen in Fig. 4, in comparison with the normal group, the other 6 groups had increased glomerular mesangial matrix, narrowed or damaged capillary lumen, adhesion of capillary plexus and cyst wall, and crescent formation in some glomeruli. There were no significant differences in the pathological changes between the blank and NC groups. Compared with the blank and NC groups, the miR-181a mimic and si-CRY1 groups both showed a distinct glomus and renal balloon structure, evenly distributed endothelial cells and mesangial cells, as well as significantly reduced degrees of pathological changes. The miR-181a inhibitor group also presented with a severely deformed glomerular balloon, shrunken capillaries, and occlusion in parts of the vascular lumen, along with swelling, disorientation, and partial loss of RTE cells. There was no significant difference in the extent of the lesion in the miR-181a inhibitor + si-CRY1 group, indicating that up-regulation of miR-181a or down-regulation of CRY1 plays a significant part in reducing the degree of CKD.

### Up-regulated miR-181a or down-regulated CRY1 reduces GSI

**Fig. 5:**
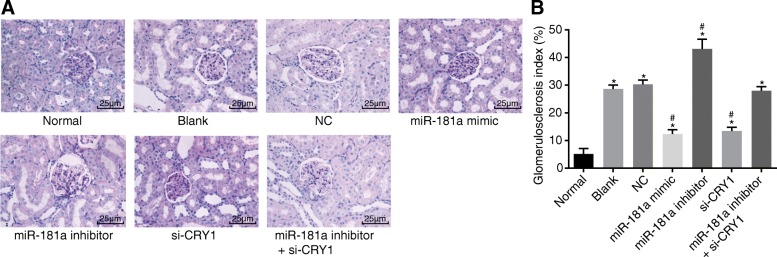
PASM staining (× 400) shows that up-regulated miR-181a or down-regulated CRY1 reduces the GSI. Notes: Panel **a**, results of PASM staining under a microscope; Panel **b**, histogram of PASM results; ^*^, *p* < 0.05 vs. the normal group; ^#^, *p* < 0.05 vs. the blank and NC groups; the data are presented as the mean ± standard deviation, analyzed by one-way ANOVA; the experiment was independently repeated three times; NC, negative control; miR-181a, microRNA-181a; CRY1, cryptochrome 1; GSI, glomerulosclerosis index; PASM, periodic Schiff-methenamine

PASM staining was used to detect the GSI (Fig. 5). No obvious lesions were found in the glomeruli of the normal rats. In comparison with the normal group, an obvious collapse and dissolution of the glomerular capillary network, expansion in certain capillaries, micro-thrombosis in the renal capillary lumen, fractures in the renal capsule, a large number of inflammatory cells infiltrating the glomerulus, and a significant increase in GSI in the 6 CKD groups were all noticeable (all *p* < 0.05). There were no obvious differences in the pathological changes between the blank and NC groups (*p* > 0.05). In comparison with both the blank and NC groups, the pathological changes found among the miR-181a mimic group and the si-CRY1 group were significantly reduced, with the GSI showing a significant decline (*p* < 0.05). The pathological changes were significantly induced and the GSI increased in the miR-181a inhibitor group (all *p* < 0.05). The pathological changes in the miR-181a inhibitor + si-CRY1 group were not significantly different (*p* > 0.05). These findings indicate that the up-regulation of miR-181a or down-regulation of CRY1 expression can reduce the GSI.

### Up-regulated miR-181a or down-regulated CRY1 reduces the degree of tubulointerstitial lesions

**Fig. 6:**
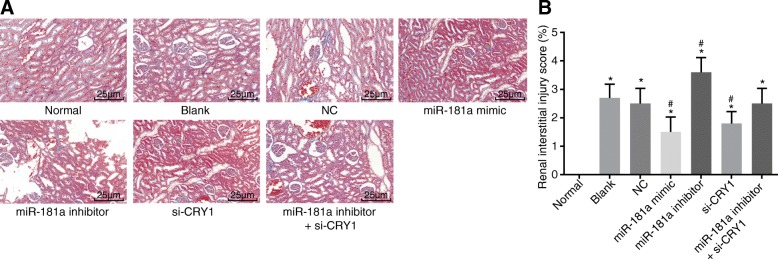
Masson staining (× 400) shows that up-regulated miR-181a or down-regulated CRY1 reduces the degree of tubulointerstitial lesions. Notes: Panel **a**, results of Masson staining under a microscope; Panel **b**, histogram of Masson results; ^*^, *p* < 0.05 vs. the normal group; ^#^, *p* < 0.05 vs. the blank and NC groups; the data are presented as the mean ± standard deviation, analyzed by one-way ANOVA; the experiment was independently repeated three times; NC, negative control; miR-181a, microRNA-181a; CRY1, cryptochrome 1

Next, we employed Masson staining to detect the degree of the tubulointerstitial lesions. As shown in Fig. 6, compared with the normal group, the remaining 6 groups showed signs of focal atrophy in the renal tubulus, renal interstitial lymphocyte infiltration, focal fibrosis, podocyte swelling, and higher levels of renal tubulointerstitial lesions (all *p* < 0.05). There was no significant difference in the degree of tubulointerstitial lesions between the blank and NC groups (*p* > 0.05). In comparison with both the blank and NC groups, the pathological changes and the degree of tubulointerstitial lesions were significantly less in both the miR-181a mimic and si-CRY1 groups, while the pathological changes were greater and the degree of tubulointerstitial lesions higher in the miR-181a inhibitor group (all *p* < 0.05). No significant differences in the pathological changes in the miR-181a inhibitor + si-CRY group were detected (*p* > 0.05). The aforementioned results indicated that the up-regulation of miR-181a or down-regulation of CRY1 expression can immensely reduce the degree of the tubulointerstitial lesions.

### Up-regulated miR-181a or down-regulated CRY1 inhibits apoptosis of RTE cell

**Fig. 7:**
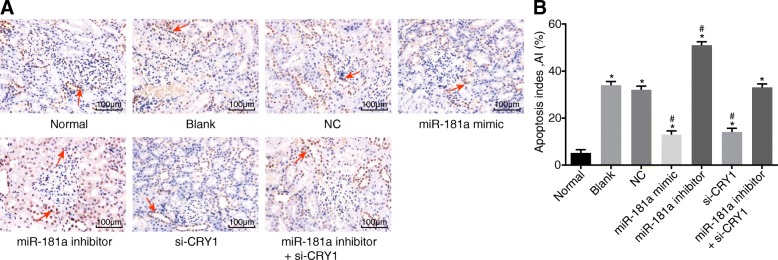
TUNEL staining indicates that RTE cell apoptosis is inhibited by overexpression of miR-181a or down-regulation of CRY1 (× 200). Notes: Panel **a**, results of TUNEL staining under a microscope; Panel **b**, histogram of TUNEL staining results; ^*^, *p* < 0.05 vs. the normal group; ^#^, *p* < 0.05 vs. the blank and NC groups; the data are presented as the mean ± standard deviation, analyzed by one-way ANOVA; the experiment was independently repeated three times; NC, negative control; miR-181a, microRNA-181a; CRY1, cryptochrome 1

RTE cell apoptosis was detected using the TUNEL staining method (Fig. 7). In comparison with the normal group, the apoptotic rate of the aforementioned apoptotic RTE cells was significantly higher in the remaining 6 groups (*p* < 0.05). No differences were found in the apoptotic rate between the blank and NC groups (*p* > 0.05). In comparison with both the blank and NC groups, the apoptotic rate of the RTE cells was considerably lower in the miR-181a mimic group and the si-CRY1 group, while it was elevated in the miR-181a inhibitor group (all *p* < 0.05). No significant differences were found in the apoptotic rate in the miR-181a inhibitor + si-CRY1 group (*p* > 0.05), indicating that either the up-regulation of miR-181a or down-regulation of CRY1 can directly inhibit the apoptosis process of RTE cells.

### CRY1 is a target gene of miR-181a

**Fig. 8:**
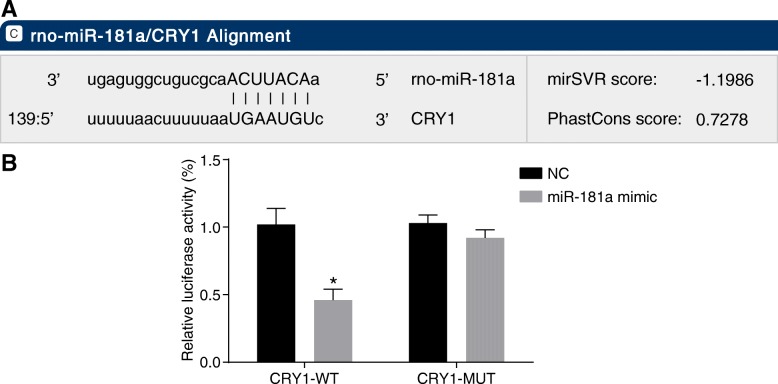
A targeting/regulatory relationship between miR-181a and CRY1 is identified by the target prediction program and determination of luciferase activity. Notes: Panel **a**, predicted binding sites for miR-181a in the CRY1 3’UTR; Panel **b**, luciferase activity of cells transfected with CRY1–3’UTR-WT and CRY1–3’UTR-MUT; ^*^, *p* < 0.05 vs. the NC group; the data are presented as the mean ± standard deviation, analyzed by *t*-test; the experiment was independently repeated three times; miR-181a, microRNA-181a; CRY1, cryptochrome 1; NC, negative control; UTR, untranslated region; WT, wild-type; MUT, mutant

According to the analysis provided by online analysis software, there was a specific binding site between the CRY1 gene and miR-181a sequence, suggesting that CRY1 is a direct target gene of miR-181a (Fig. 8a). A dual luciferase reporter gene assay was used to verify this prediction. As depicted in Fig. 8b, the luciferase activity of the cells transfected with wild-type (Wt)-miR-181a/CRY1 in the miR-181a mimic group was significantly lower in comparison to the NC group (*p* < 0.05). No significant differences were observed, however, in the luciferase activity of the mutant (Mut) 3’UTR (*p* > 0.05). These results indicate that miR-181a can specifically bind to CRY1 and down-regulate the gene expression.

### Up-regulated miR-181a or down-regulated CRY1 suppresses the activation of the TLR/NF-κB pathway by decreasing the mRNA expression of TLR/NF-κB pathway-related genes

**Fig. 9:**
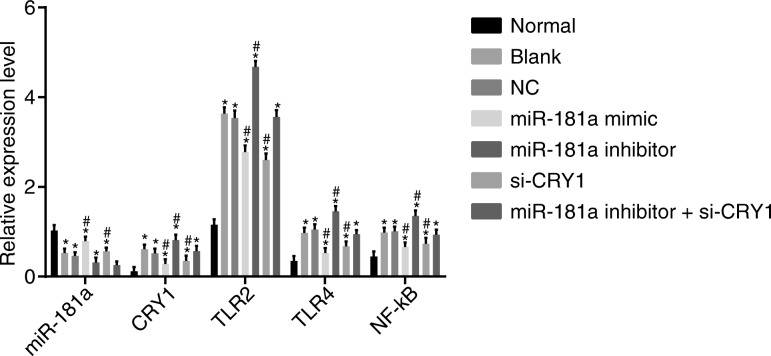
RT-qPCR results demonstrate that up-regulated miR-181a or down-regulated CRY1 suppresses the activation of the TLR/NF-κB pathway by decreasing the mRNA expression of TLR/NF-κB pathway-related genes. Notes: ^*^, *p* < 0.05 vs. the normal group; ^#^, *p* < 0.05 vs. the blank and NC groups; the data are presented as the mean ± standard deviation, analyzed by one-way ANOVA; the experiment was independently repeated three times; NC, negative control; miR-181a, microRNA-181a; CRY1, cryptochrome 1; TLR2, Toll-like receptor 2; TLR4, Toll-like receptor 4; NF-κB, nuclear factor-kappa B; CKD, chronic kidney disease

The effect of miR-181a on the TLR/NF-κB pathway was analyzed using RT-qPCR. The results of the RT-qPCR are illustrated in Fig. 9. A notable increase in CRY1, TLR2, TLR4, and NF-κB mRNAs and a significant decrease in miR-181a expression in the 6 CKD groups were seen compared with the normal group (all *p* < 0.05). No significant difference was observed in miR-181a, CRY1, TLR2, TLR4, or NF-κB expression between the blank and NC groups (*p* > 0.05). The miR-181a mimic and si-CRY1 groups had reduced mRNA expression of CRY1, TLR2, TLR4, and NF-κB compared to the blank and NC groups (*p* < 0.05). miR-181a was up-regulated in the miR-181a mimic group (*p* < 0.05) while exhibiting no significant differences in the si-CRY1 group (*p* > 0.05). The mRNA changes in the miR-181a inhibitor group were opposite to those of the miR-181a mimic group. In the miR-181a inhibitor + si-CRY1 group, the expression of miR-181a was decreased (*p* < 0.05), with no significant differences in other indexes, compared to both the blank and NC groups (*p* > 0.05). These results show that the overexpression of miR-181a along with the silencing of CRY1 expression can suppress the activation of the TLR/NF-κB pathway.

### Up-regulated miR-181a or down-regulated CRY1 inhibits the activation of the TLR/NF-κB pathway by decreasing the protein expression of TLR/NF-κB pathway-related genes

**Fig. 10:**
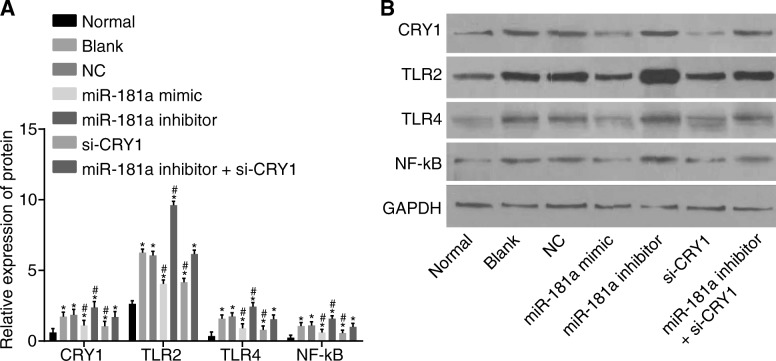
Western blot reveals that up-regulated miR-181a or down-regulated CRY1 inhibits the activation of the TLR/NF-κB pathway by decreasing the protein expression of TLR/NF-κB pathway-related genes. Notes: Panel **a**, bar graph of protein expression of CRY1, TLR2, TLR4, and NF-κB in each group; Panel **b**, protein bands of CRY1, TLR2, TLR4, and NF-κB in each group; ^*^, *p* < 0.05 vs. the normal group; ^#^, *p* < 0.05 vs. the blank and NC groups; the data are presented as the mean ± standard deviation, analyzed by one-way ANOVA; the experiment was independently repeated three times; NC, negative control; miR-181a, microRNA-181a; CRY1, cryptochrome 1; TLR2, toll-like receptor 2; TLR4, toll-like receptor 4; NF-κB, nuclear factor-kappa B; CKD, chronic kidney disease

To further investigate whether miR-181a or CRY1 could affect the TLR/NF-κB pathway, CRY1, TLR2, TLR4, and NF-κB were detected using the western blot assay (Fig. 10). In comparison with the normal group, the CRY1, TLR2, TLR4, and NF-κB proteins were significantly elevated in the other 6 groups (all *p* < 0.05). All four proteins were similar between the blank and NC groups (*p* > 0.05). Compared with the blank and NC groups, all of those proteins were significantly decreased in both the miR-181a mimic and si-CRY1 groups, while they were significantly increased in the miR-181a inhibitor group (all *p* < 0.05). No significant differences were detected in the miR-181a inhibitor + si-CRY1 group (*p* > 0.05). These results indicate that the overexpression of miR-181a or silencing of CRY1 expression can inhibit the activation of the TLR/NF-κB pathway.

## Discussion

The inflammatory responses occurring during the progression of the damage of renal tissue as well as any tissue-related injury caused by CKD might develop into end-stage renal disease (Liu et al. 2013). miRNAs are important biomarkers in the diagnosis, prognosis, and prediction of renal development and disease (Neal et al. 2011; Xu et al. 2012). Based on these findings, the present study was conducted to investigate the effects of miR-181a and CRY1 on GS and RTE injury in CKD. Our results provide evidence that up-regulating miR-181a potentially suppresses the expression of CRY1 along with the activation of the TLR/NF-κB, thereby making a significant contribution to the alleviation of both GS and RTE injury of CKD.

Initially, our study revealed that miR-181a decreased the 24-h urinary protein levels of CKD and ameliorated its renal damage, which was reflected in the decreased contents of albumin, uric acid, urea, serum urea nitrogen, and serum creatinine after transfection. Serum albumin, uric acid, serum urea nitrogen and serum creatinine can be used as parameters to evaluate CKD progression (Li et al. 2014). Up-regulating miR-181c also protects kidneys from CsA-induced renal injury and fibrosis through the suppression of the epithelial-mesenchymal transition (EMT) (Sun et al. 2017). Consistent with those findings, our study found that miR-181a inhibited both the GS and RTE injury in CKD, which was supported by an apparent decrease in the cell apoptosis rate and the levels of ROS, MDA, IL-1β, IL-6, and TNF-α in the miR-181a mimic group. In addition, the overexpression of miR-181a plays important roles in renal disease by inhibiting the cisplatin-induced apoptosis of tubular epithelial cells (Zhu et al. 2012). Another study reported that miR-181c suppresses the downstream production of pro-inflammatory mediators, such as TNF-α, IL-1β, and iNOS (Zhang et al. 2015). Moreover, the up-regulation of miR-181a further inhibits IL-6 and TNF-α in dendritic cells by targeting c-Fos (Wu et al. 2012). Although the aforementioned findings suggest that miR-181a could be a potentially important target in the treatment of CKD, further studies on its underlying mechanism were needed.

miRNAs affect the behaviors of malignant cells by silencing a variety of target genes, and regulating the downstream signaling pathways (Zhao et al. 2016). Our study provided evidence that CRY1 is indeed a target gene of miR-181a and that the elevation of miR-181a could lead to the direct down-regulation of CRY1. Moreover, miR-181d expression can also directly target the 3’-UTRs of CRY2 and FBXL3, providing information on its association with colorectal cancer (Guo et al. 2017). Our results and others’ lead to the conclusion that an increased expression of miR-181a results in the down-regulation of CRY1, in turn promoting the GS and RTE injury in CKD.

NF-κB, a general nuclear transcription factor consisting of two glutenin subunits (p65 and p50), acts at the center of the inflammatory response and controls the gene expression of numerous inflammation-associated substances, including inflammatory cytokines (IL-1β, IL-6, IL-8, and TNF-α), as well as genes involved in ROS production (Jiang et al. 2014). Inhibiting the activation of the TLR4/NF-κB pathway reduces uric acid-induced EMT and inflammatory cytokine expression in the HK-2 cells of hyperuricemia nephropathy (Liu et al. 2017). Our findings show that TLR/NF-κB pathway-related proteins and inflammatory cytokines (IL-1β, IL-6 and TNF-α) were significantly reduced in both the miR-181a mimic and si-CRY1 groups, and over-expression of miR-181a seemed to reduce the GS and RTE injury by inhibiting the TLR/NF-κB pathway. Additionally, the inhibition of the TLR/NF-κB pathway by miR-181a could reduce inflammation in coronary artery disease (Hulsmans et al. 2012). Narasimamurthy et al. ascertained that the down-regulation of CRY, a core clock gene component, activates pro-inflammatory cytokines via the NF-κB pathway in chronic inflammatory disease (Narasimamurthy et al. 2012). There have also been various experimental and clinical data reports indicating TLRs are directly involved in the pathogenesis of urinary tract infections, acute kidney injury, and lupus nephritis (Chen et al. 2011; Ding et al. 2015; Moreth et al. 2014). We conclude that the up-regulation of miR-181a or down-regulation of CRY1 is directly associated with the inhibition of the TLR/NF-κB pathway, which reduces both the GS and RTE injury in CKD.

## Conclusion

The overexpression of miR-181a could suppress both GS and RTE injury in patients diagnosed with CKD by down-regulating the expression of CRY1 via inhibition of the TLR/NF-κB pathway. Although the exact underlying mechanism of the regulation of the CKD metabolic pathway remains largely unknown, we believe that our findings improve our understanding of the specific mechanism by which the miR-181a-targeted CRY1 has an unfavorable effect on RTE cells in CKD via the TLR/NF-κB pathway.

## Abbreviations

ANOVA: Analysis of variance
BCA: Bicinchoninic acid
BSA: Bovine serum albumin
CKD: Chronic kidney disease
CRY1: Cryptochrome 1
CVD: Cardiovascular disease
ECL: Electrochemiluminescence
ELISA: Enzyme-linked immunosorbent assay
EMT: Epithelial-mesenchymal transition
GSI: Glomerulosclerosis index
HE: Hematoxylin-eosin
HRP: Horseradish peroxidase
miRNA: microRNA
Mut: Mutant
NC: Negative control
NC: Nitrocellulose
OD: Optical density
PAGE: Polyacrylamide gel electrophoresis
PASM: Periodic acid of Schiff-methenamine
PBS: Phosphate-buffered saline
RTE: Renal tubular epithelial
RT-qPCR: Reverse transcription–quantitative polymerase chain reaction
SD: Sprague-Dawley
SPSS: Statistical Package for the Social Sciences
TDT: Terminal deoxyribonucleotidyl transferase
Wt: Wild-type

## References

[CR1] Chen J, John R, Richardson JA, Shelton JM, Zhou XJ, Wang Y (2011). Toll-like receptor 4 regulates early endothelial activation during ischemic acute kidney injury. Kidney Int.

[CR2] Ding LH, Liu D, Xu M, Wu M, Liu H, Tang RN (2015). TLR2-MyD88-NF-kappaB pathway is involved in tubulointerstitial inflammation caused by proteinuria. Int J Biochem Cell Biol.

[CR3] Ding ZH, Xu LM, Wang SZ, Kou JQ, Xu YL, Chen CX (2014). Ameliorating Adriamycin-induced chronic kidney disease in rats by orally administrated Cardiotoxin from Naja naja atra venom. Evid Based Complement Alternat Med.

[CR4] Gaddam S, Gunukula SK, Lohr JW, Arora P (2016). Prevalence of chronic kidney disease in patients with chronic obstructive pulmonary disease: a systematic review and meta-analysis. BMC Pulm Med.

[CR5] Gandolfo MT, Verzola D, Salvatore F, Gianiorio G, Procopio V, Romagnoli A (2004). Gender and the progression of chronic renal diseases: does apoptosis make the difference?. Minerva Urol Nefrol.

[CR6] Guo X, Zhu Y, Hong X, Zhang M, Qiu X, Wang Z (2017). miR-181d and c-myc-mediated inhibition of CRY2 and FBXL3 reprograms metabolism in colorectal cancer. Cell Death Dis.

[CR7] Hulsmans M, Sinnaeve P, Van der Schueren B, Mathieu C, Janssens S, Holvoet P (2012). Decreased miR-181a expression in monocytes of obese patients is associated with the occurrence of metabolic syndrome and coronary artery disease. J Clin Endocrinol Metab.

[CR8] Jiang GT, Chen X, Li D, An HX, Jiao JD (2014). Ulinastatin attenuates renal interstitial inflammation and inhibits fibrosis progression in rats under unilateral ureteral obstruction. Mol Med Rep.

[CR9] Lee KH, Kim SH, Lee HR, Kim W, Kim DY, Shin JC (2013). MicroRNA-185 oscillation controls circadian amplitude of mouse Cryptochrome 1 via translational regulation. Mol Biol Cell.

[CR10] Lei Z, Ma X, Li H, Zhang Y, Gao Y, Fan Y, et al. Up-regulation of miR-181a in clear cell renal cell carcinoma is associated with lower KLF6 expression, enhanced cell proliferation, accelerated cell cycle transition, and diminished apoptosis. Urol Oncol. 2017; 10.1016/j.urolonc.2017.09.019 29066014

[CR11] Levey AS, Coresh J (2012). Chronic kidney disease. Lancet.

[CR12] Levey AS, Eckardt KU, Tsukamoto Y, Levin A, Coresh J, Rossert J (2005). Definition and classification of chronic kidney disease: a position statement from kidney disease: improving global outcomes (KDIGO). Kidney Int.

[CR13] Li L, Chang A, Rostand SG, Hebert L, Appel LJ, Astor BC (2014). A within-patient analysis for time-varying risk factors of CKD progression. J Am Soc Nephrol.

[CR14] Liu H, Sun W, Wan YG, Tu Y, Yu BY, Hu H (2013). Regulatory mechanism of NF-kappaB signaling pathway on renal tissue inflammation in chronic kidney disease and interventional effect of traditional Chinese medicine. Zhongguo Zhong Yao Za Zhi.

[CR15] Liu H, Xiong J, He T, Xiao T, Li Y, Yu Y (2017). High uric acid-induced epithelial-mesenchymal transition of renal tubular epithelial cells via the TLR4/NF-kB signaling pathway. Am J Nephrol.

[CR16] Lorenzen JM, Haller H, Thum T (2011). MicroRNAs as mediators and therapeutic targets in chronic kidney disease. Nat Rev Nephrol.

[CR17] Marques FZ, Romaine SP, Denniff M, Eales J, Dormer J, Garrelds IM, et al. Signatures of miR-181a on renal transcriptome and blood pressure. Mol Med. 2015; 10.2119/molmed.2015.00096 PMC4818264 26322847

[CR18] Moreth K, Frey H, Hubo M, Zeng-Brouwers J, Nastase MV, Hsieh LT (2014). Biglycan-triggered TLR-2- and TLR-4-signaling exacerbates the pathophysiology of ischemic acute kidney injury. Matrix Biol.

[CR19] Munoz-Felix JM, Oujo B, Lopez-Novoa JM (2014). The role of endoglin in kidney fibrosis. Expert Rev Mol Med.

[CR20] Na YJ, Sung JH, Lee SC, Lee YJ, Choi YJ, Park WY (2009). Comprehensive analysis of microRNA-mRNA co-expression in circadian rhythm. Exp Mol Med.

[CR21] Narasimamurthy R, Hatori M, Nayak SK, Liu F, Panda S, Verma IM (2012). Circadian clock protein cryptochrome regulates the expression of proinflammatory cytokines. Proc Natl Acad Sci U S A.

[CR22] Neal CS, Michael MZ, Pimlott LK, Yong TY, Li JY, Gleadle JM (2011). Circulating microRNA expression is reduced in chronic kidney disease. Nephrol Dial Transplant.

[CR23] O'Hare AM, Choi AI, Bertenthal D, Bacchetti P, Garg AX, Kaufman JS (2007). Age affects outcomes in chronic kidney disease. J Am Soc Nephrol.

[CR24] Sampaio-Maia B, Simoes-Silva L, Pestana M, Araujo R, Soares-Silva IJ (2016). The role of the gut microbiome on chronic kidney disease. Adv Appl Microbiol.

[CR25] Su R, Lin HS, Zhang XH, Yin XL, Ning HM, Liu B (2015). MiR-181 family: regulators of myeloid differentiation and acute myeloid leukemia as well as potential therapeutic targets. Oncogene.

[CR26] Sun W, Min B, Du D, Yang F, Meng J, Wang W (2017). miR-181c protects CsA-induced renal damage and fibrosis through inhibiting EMT. FEBS Lett.

[CR27] Uil M, Scantlebery AML, Butter LM, Larsen PWB, de Boer OJ, Leemans JC (2018). Combining streptozotocin and unilateral nephrectomy is an effective method for inducing experimental diabetic nephropathy in the ‘resistant’ C57Bl/6J mouse strain. Sci Rep.

[CR28] Wu C, Gong Y, Yuan J, Zhang W, Zhao G, Li H (2012). microRNA-181a represses ox-LDL-stimulated inflammatory response in dendritic cell by targeting c-Fos. J Lipid Res.

[CR29] Xu J, Li R, Workeneh B, Dong Y, Wang X, Hu Z (2012). Transcription factor FoxO1, the dominant mediator of muscle wasting in chronic kidney disease, is inhibited by microRNA-486. Kidney Int.

[CR30] Yang L, Chu Y, Wang L, Wang Y, Zhao X, He W (2015). Overexpression of CRY1 protects against the development of atherosclerosis via the TLR/NF-kappaB pathway. Int Immunopharmacol.

[CR31] Zhang L, Li YJ, Wu XY, Hong Z, Wei WS (2015). MicroRNA-181c negatively regulates the inflammatory response in oxygen-glucose-deprived microglia by targeting toll-like receptor 4. J Neurochem.

[CR32] Zhao X, Zhou Y, Chen YU, Yu F (2016). miR-494 inhibits ovarian cancer cell proliferation and promotes apoptosis by targeting FGFR2. Oncol Lett.

[CR33] Zhu HY, Liu MY, Hong Q, Zhang D, Geng WJ, Xie YS (2012). Role of microRNA-181a in the apoptosis of tubular epithelial cell induced by cisplatin. Chin Med J (Engl).

